# Comparative Efficacy of Extracorporeal Versus Conventional Cardiopulmonary Resuscitation in Adult Refractory Out-of-Hospital Cardiac Arrest: A Retrospective Study at a Single Center

**DOI:** 10.3390/jcm14020513

**Published:** 2025-01-15

**Authors:** Juncheol Lee, Yong Ho Jeong, Yun Jin Kim, Yongil Cho, Jaehoon Oh, Hyo Jun Jang, Yonghoon Shin, Ji Eon Kim, Hee Jung Kim, Yang Hyun Cho, Jae Seung Jung, Jun Ho Lee

**Affiliations:** 1 Department of Emergency Medicine, Hanyang University College of Medicine, Seoul 04763, Republic of Korea; doldoly@hanyang.ac.kr (J.L.); joeguy@hanyang.ac.kr (Y.C.); ojjai@hanyang.ac.kr (J.O.); 2 Department of Thoracic and Cardiovascular Surgery, Kangnam Sacred Heart Hospital, Hallym University Medical Center, Hallym University College of Medicine, Seoul 07441, Republic of Korea; fauntleroy@hallym.or.kr; 3 Department of Medicine, Hanyang University College of Medicine, Seoul 04763, Republic of Korea; yeun0148@hanyang.ac.kr; 4 Biostatistics Lab, Medical Research Collaborating Center, Hanyang University, Seoul 04763, Republic of Korea; 5 Department of Thoracic and Cardiovascular Surgery, Hanyang University Seoul Hospital, Hanyang University College of Medicine, Seoul 04763, Republic of Korea; rgo38@hanyang.ac.kr; 6 Department of Thoracic and Cardiovascular Surgery, Korea University Anam Hospital, Korea University College of Medicine, 73 Goryeodae-ro, Seongbuk-gu, Seoul 02841, Republic of Korea; peaceful_heart@korea.ac.kr (Y.S.); 19800@korea.ac.kr (J.E.K.); berserking@korea.ac.kr (H.J.K.); heartistcs@korea.ac.kr (J.S.J.); 7 Department of Thoracic and Cardiovascular Surgery, Samsung Medical Center, Sungkyunkwan University College of Medicine, Seoul 06351, Republic of Korea; yanghyun.cho@samsung.com

**Keywords:** out-of-hospital cardiac arrest, extracorporeal cardiopulmonary resuscitation, conventional cardiopulmonary resuscitation, neurological outcome

## Abstract

**Background:** Extracorporeal cardiopulmonary resuscitation (ECPR) has the potential to improve neurological outcomes in patients with refractory out-of-hospital cardiac arrest (OHCA), offering an alternative to conventional cardiopulmonary resuscitation (CCPR). However, its effectiveness in OHCA remains controversial despite advancements in resuscitation techniques. **Methods:** This retrospective single-center study compared neurological outcomes and 30-day survival between ECPR and CCPR patients from January 2014 to January 2022. Patients aged 18–75 with witnessed OHCA, minimal no flow and low flow times, and cardiac arrests occurring at home or in public places were included. All patients were transported directly to our institution, a tertiary medical center serving the southeastern region of Seoul, where extracorporeal membrane oxygenation implantation was consistently performed in the emergency department. Neurological outcomes were assessed using Cerebral Performance Category scores, with good outcomes defined as scores of 1–2. Statistical analyses included logistic regression models and Kaplan–Meier survival curves, adjusted for confounders using inverse probability of treatment weighting. **Results:** ECPR was associated with significantly better neurological outcomes than CCPR (*p* < 0.001). Factors predicting poor outcomes included older age and longer low flow times, while male sex and shockable rhythms were protective. No significant difference was found in 30-day survival between the ECPR and CCPR groups, although a trend toward better survival was noted with ECPR. **Conclusions:** ECPR may improve neurological outcomes in patients with refractory OHCA compared to CCPR, although it does not significantly affect 30-day survival. Further studies are necessary to validate these findings and explore the long-term impacts of ECPR.

## 1. Introduction

Survival with good neurological outcomes after out-of-hospital cardiac arrest (OHCA) has improved significantly in recent decades [1,2]. Key factors contributing to this improvement include the increased use of bystander cardiopulmonary resuscitation (CPR) and early defibrillation in the pre-hospital phase, changes in medications administered during CPR, and advancements in post-cardiac arrest interventions such as targeted temperature management [3,4,5]. Additionally, smartphone-based activation of community first responders has demonstrated life-saving potential and gained considerable attention across Europe [6]. However, despite these improvements, OHCA remains a major public health challenge and a leading cause of mortality and morbidity [2,3,4,5,6,7].

Recently, veno-arterial extracorporeal membrane oxygenation (ECMO) was introduced during cardiopulmonary resuscitation (CPR), a technique known as extracorporeal cardiopulmonary resuscitation (ECPR) [8,9]. ECPR is not suitable for all patients; it is typically indicated for those with suspected cardiac origins, such as refractory shockable rhythms. Ideal candidates often include relatively younger patients with short no flow and low flow times, such as witnessed arrests or those receiving bystander CPR. ECPR can provide higher global blood flow compared to conventional CPR (CCPR) and may help reduce anoxic cellular damage in post-cardiac arrest patients, potentially mitigating ischemia–reperfusion injury [10,11]. Additionally, while CCPR cannot address reversible cardiac causes, such as those treated by percutaneous coronary intervention or open-heart surgery, ECPR can act as a bridge to enable these interventions in cardiac arrest patients.

Numerous studies have compared the effectiveness of ECPR and CCPR, yet the outcomes for ECPR patients remain controversial [11,12,13,14,15,16,17,18]. While several studies suggest that ECPR improves survival rates in OHCA patients compared to CCPR [11,12,13,14,15], other research has found no significant impact on survival rates or neurological outcomes, highlighting insufficient evidence for its overall effectiveness [16,17,18]. Recently, there has been an increasing focus on conducting meta-analyses of ECPR in OHCA patients [19,20,21]. Wang and colleagues reported a lack of high-quality evidence to confirm the superiority of ECPR over CCPR in improving neurological outcomes for OHCA patients [19]. Conversely, other studies have shown improvements in both the survival and neurological outcomes associated with ECPR [20,21]. However, significant heterogeneity has been noted among the included studies and the outcomes reported [20]. To address this, our study aimed to compare the neurological outcomes and 30-day survival of patients who received ECPR and CCPR.

## 2. Materials and Methods

### 2.1. Study Patients

**Figure 1:**
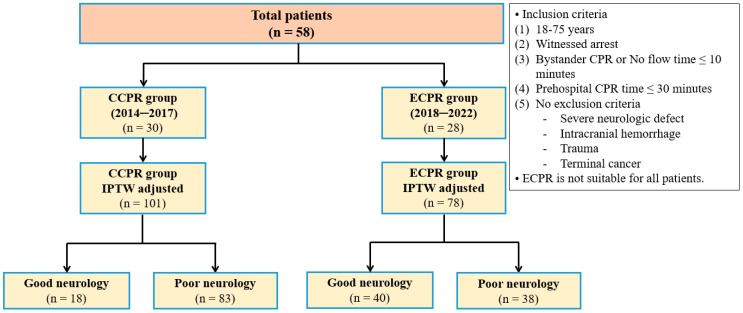
Flow diagram. CCPR, conventional cardiopulmonary resuscitation; ECPR, extracorporeal cardiopulmonary resuscitation; IPTW, inverse probability of treatment weighting.

This retrospective single-center study was conducted in the emergency room (ER) of Hanyang University Seoul Hospital, including patients who experienced OHCA and received CPR between January 2014 and January 2022 (Figure 1). Since 2018, our institution has actively utilized ECMO, and ECPR has been performed on cardiac arrest patients who meet the criteria for ECMO. We defined the group that received CPR without ECMO between 2014 and 2017, before ECMO was actively utilized, as the CCPR group, and the group that received CPR with ECMO from 2018 onwards as the ECPR group.

The study included adult patients aged 18 to 75 years who experienced cardiac arrest at home or in public places. Eligible patients received immediate bystander CPR with a no flow time of less than 10 min and underwent CPR for less than 30 min prior to hospital arrival. All patients were transported directly to our institution, a tertiary medical center serving the southeastern region of Seoul, where ECMO implantation was consistently performed in the emergency department. ECMO implantation was considered for patients with refractory cardiac arrest who did not achieve a return of spontaneous circulation through CCPR [22], including defibrillation or medication administration (e.g., epinephrine), either upon hospital arrival or shortly thereafter. Patients with severe neurological defects, intracranial hemorrhage, trauma, or terminal cancer were excluded.

In South Korea, all ECMO implantations are performed after patients arrive at the emergency department of hospitals. Unlike in a few other countries [9], a pre-hospital or on-scene ECMO insertion system has not yet been established. In our study, the duration of pre-hospital CPR, including chest compressions, was documented by EMS. For patients undergoing ECPR, the in-hospital CPR duration was defined as the time interval from hospital arrival to the initiation of ECMO. Low flow time was defined as the combined total duration of pre-hospital and in-hospital CPR.

### 2.2. Study Outcomes

A retrospective review of medical records was conducted to assess the outcomes of the OHCA patients. The primary outcome was the neurological outcomes during hospitalization. Neurological outcomes were categorized based on Cerebral Performance Category (CPC) scores, with scores of 1 (representing good cerebral performance) and 2 (indicating moderate cerebral disability) considered good neurological outcomes. Conversely, scores of 3 (representing severe cerebral disability) to 5 (indicating brain death) were categorized as poor neurological outcomes [23]. The CPC score assessment was performed with double verification by both neurologists and emergency medicine specialists. The secondary outcome analyzed was 30-day survival.

### 2.3. Statistical Analysis

Continuous variables were presented as medians with interquartile ranges (IQRs) when skewed, as determined by the Kolmogorov–Smirnov test. The Wilcoxon rank-sum test was used to compare continuous variables. Categorical variables were expressed as frequencies and percentages, with comparisons made using the chi-square test or Fisher’s exact test, as appropriate. The chi-square test was employed to compare categorical variables between the two groups. Univariable and multivariable logistic regression models were used to identify independent predictors of poor neurological outcomes, with a forest plot generated based on the results of the multivariable analysis. Significant variables from the univariable analyses were included in the multivariable models. Kaplan–Meier survival curves were estimated to assess 30-day survival, and the log-rank test was used to compare survival rates between the two groups.

To adjust for baseline differences, we used weighted logistic regression models with inverse probability of treatment weighting (IPTW) to minimize potential confounding factors [24]. The adjusted variables included age, sex, bystander CPR, first documented arrest rhythm by emergency medical services (EMS), no flow time, and low flow time. For continuous and dichotomous variables, the standardized mean differences were calculated using the mean and prevalence, respectively (Table 1).

All tests were two-tailed, and statistical significance was set at a *p* value of less than 0.05. Statistical analyses were performed using R software (version 3.5.1; R Foundation for Statistical Computing, Vienna, Austria) and SAS software (version 9.4; SAS Institute Inc., Cary, NC, USA).

## 3. Results

### 3.1. Baseline Characteristics of All Patients

Table 1 summarizes the baseline characteristics of the ECPR and CCPR patients. In the unadjusted cohort, the median age of all patients was 58.0 years (IQR, 50.0–68.0), with 79.3% being male. The most common cause of cardiac arrest was acute myocardial infarction, occurring in 15 patients (53.6%) in the ECPR group and 10 patients (33.3%) in the CCPR group. Most of the cardiac arrests were witnessed by laypersons (82.8%), and bystander CPR was performed in 60.3% of the cases. Shockable arrhythmias, including ventricular fibrillation and pulseless ventricular tachycardia, were the most frequently detected rhythms by EMS and ER personnel. The median no flow time was 1.0 min (IQR, 0.0–5.0), and the median low flow time was 45.5 min (IQR, 32.0–57.0). The baseline characteristics of the IPTW-adjusted cohort are also summarized in Table 1.

### 3.2. Characteristics of Patients According to Neurological Outcomes

**Figure 2:**
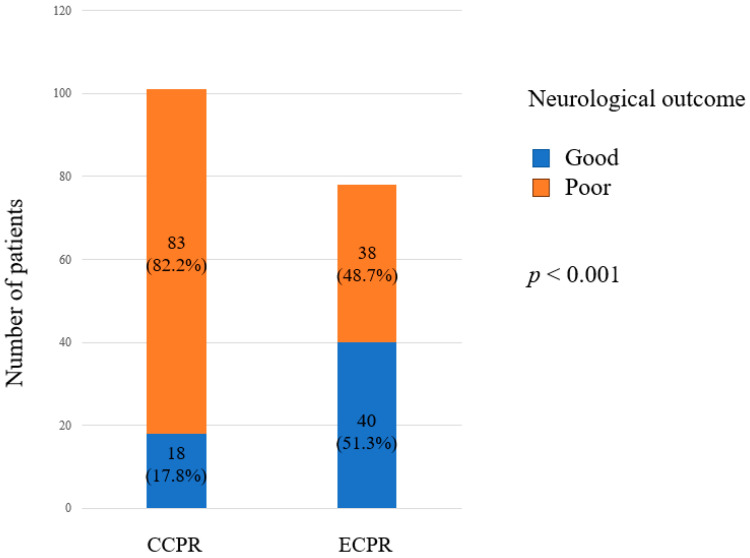
Distribution of good and poor neurological outcomes in CCPR and ECPR groups within the IPTW-adjusted cohort. CCPR, conventional cardiopulmonary resuscitation; ECPR, extracorporeal cardiopulmonary resuscitation; IPTW, inverse probability of treatment weighting.

Table S1 summarizes the characteristics of the patients according to neurological outcomes. The distribution of good and poor neurological outcomes in the CCPR and ECPR groups within the IPTW-adjusted cohort is illustrated in Figure 2. Both in the unadjusted and IPTW-adjusted cohort, CCPR was associated with a higher incidence of poor neurological outcomes (*p* = 0.0497 in the unadjusted cohort; *p* < 0.001 in the IPTW-adjusted cohort). In the IPTW-adjusted cohort, patients with good neurological outcomes were younger than those with poor neurological outcomes (52.0 vs. 66.0 years, *p* < 0.001). Additionally, the duration of low flow time was shorter in patients with good neurological outcomes compared to those with poor neurological outcomes (34.0 vs. 47.0 min, *p* = 0.034).

### 3.3. Impact of ECPR and Predictors of Poor Neurological Outcomes

**Figure 3:**
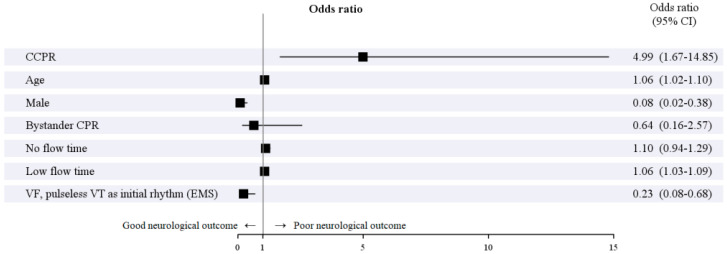
The forest plot based on the results of multivariable analysis of poor neurological outcomes after adjustment using IPTW. IPTW, inverse probability of treatment weighting; CI, confidence interval; CCPR, conventional cardiopulmonary resuscitation; CPR, cardiopulmonary resuscitation; VF, ventricular fibrillation; VT, ventricular tachycardia; EMS, emergency medical services.

The forest plot based on the multivariable analysis of poor neurological outcomes, adjusted using IPTW, is shown in Figure 3. The results of the univariable and multivariable analyses of poor neurological outcomes, also adjusted using IPTW, are summarized in Table S2. CCPR was identified as an independent predictor of poor neurological outcomes (*p* = 0.004; odds ratio [OR], 4.99; 95% confidence interval [CI], 1.67–14.85).

Older age (*p* = 0.002; OR, 1.06; 95% CI, 1.02–1.10) and the longer duration of low flow time (*p* < 0.001; OR, 1.06; 95% CI, 1.03–1.09) were also significant prognostic factors for poor neurological outcomes. In contrast, male sex (*p* = 0.002; OR, 0.08; 95% CI, 0.02–0.38) and the presence of shockable arrhythmias (e.g., ventricular fibrillation and pulseless ventricular tachycardia as initial rhythm; *p* = 0.008; OR, 0.23; 95% CI, 0.08–0.68) were significant protective factors against poor neurological outcomes.

### 3.4. 30-Day Survival in the ECPR Group Compared with Those in the CCPR Group

**Figure 4:**
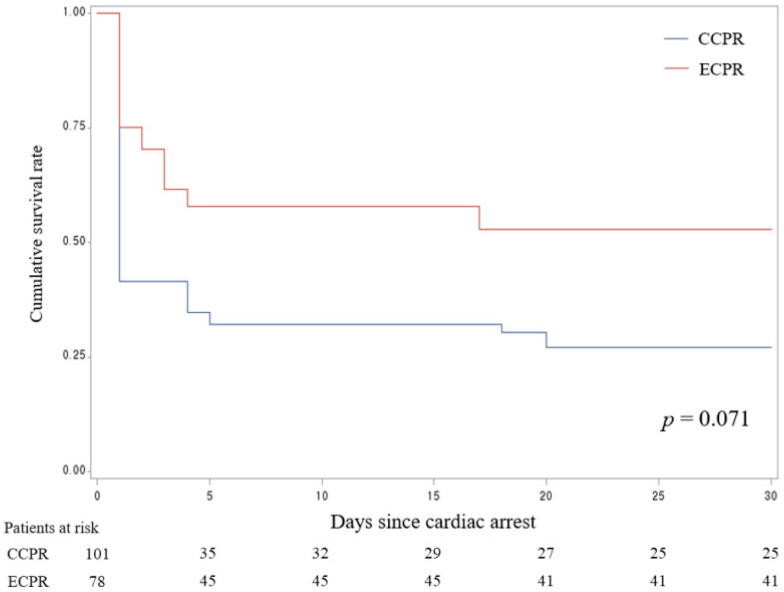
Kaplan–Meier survival curves for 30-day survival in patients with out-of-hospital cardiac arrest. CCPR, conventional cardiopulmonary resuscitation; ECPR, extracorporeal cardiopulmonary resuscitation.

Kaplan–Meier survival curves for 30-day survival in OHCA patients, adjusted using IPTW, are shown in Figure 4. While the ECPR group showed a trend toward better 30-day survival, the difference was not statistically significant compared to the CCPR group (*p* = 0.071). A subgroup analysis was conducted for patients under 60 and those 60 and older, but no significant differences were observed (Figure S1).

## 4. Discussion

In this study, we were able to determine the following: (a) CCPR was a significant predictor of poor neurological outcomes in OHCA patients; (b) older age and the longer duration of low flow time were also identified as risk factors for poor neurological outcomes; (c) male sex and the presence of shockable arrhythmias, such as ventricular fibrillation and pulseless ventricular tachycardia, were significant protective factors against poor neurological outcomes; and (d) although the ECPR group showed a trend toward better 30-day survival, the difference was not statistically significant compared to the CCPR group.

In our study, patients treated with ECPR demonstrated significantly better neurological outcomes compared to those receiving CCPR, with 51.3% of ECPR patients achieving favorable neurological outcomes (*p* < 0.001; Figure 2). This aligns with previous research, which has consistently shown superior neurological outcomes with ECPR in both short- and long-term settings [12,14,25,26]. For instance, Patricio et al. reported a nearly 50% relative increase in favorable neurological outcomes in the ECPR group compared to the CCPR group [12]. However, conflicting results have been reported in other studies; for example, Kruse et al. discussed potential patient burdens associated with ECPR [27], and a multi-center randomized trial found no significant improvement in short-term survival with favorable neurological outcomes in OHCA patients treated with ECPR [28]. The favorable neurological outcomes observed in our results may be partly due to our institution’s status as a regional emergency medical center, which demands a higher standard of medical care and facilitates early communication with EMS, potentially enhancing patient preparation and outcomes.

Several factors influence the neurological outcomes of cardiac arrest patients. In our study, older age and prolonged low flow time were identified as risk factors for poor neurological outcomes, while male sex and the presence of an initial shockable rhythm emerged as protective factors (Figure 3). Prolonged low flow time is a well-documented risk factor for survival in ECPR patients [29,30,31,32]. For instance, Shin et al. identified that low flow times under 35 min were associated with favorable neurological outcomes [31], and Shoji et al. found that a low flow time of less than 40 min was linked to improved neurological outcomes [32].

The presence of an initial shockable rhythm has also been widely recognized as a predictor of favorable neurological outcomes in cardiac arrest patients [33,34,35]. Kiyohara et al. reported that public access defibrillation, which facilitates earlier shock delivery, significantly improves survival rates and neurological outcomes [36]. In South Korea, where emergency medical services are highly developed, emergency medical technicians typically arrive at the scene within an average of nine minutes, and in urban areas, often less than five minutes [37]. This rapid response time may enhance the protective effect of shockable rhythms on neurological outcomes compared to other countries.

In contrast, although our study identified male sex as a prognostic factor for good neurological outcomes, previous studies have shown mixed results regarding the impact of sex on neurological outcomes [38,39,40]. Ng et al. found that males had a tendency to have better neurological outcomes; however, this advantage was not observed when considering younger women aged 18–44 years [39]. Our data showed a median age of 58.0 years, indicating that the predominance of post-menopausal women in our study population may have contributed to the better neurological outcomes observed in males.

In our results, the 1-month survival rate was 52.6% for ECPR patients compared to 24.8% for CCPR patients. Chen et al. reported 1-month survival rates of 34.8% for ECPR and 17.4% for CCPR, demonstrating that our results are not inferior [30]. Our institution’s approach favored the use of ECPR in younger patients, but did not impose a strict age limit. Goto et al. suggested that advanced age, particularly in patients over 75 years, is associated with reduced survival rates in ECPR patients [41]. Similarly, Lunz et al. found that stricter ECPR criteria, including restricting treatment to patients under 60, improved survival rates [42]. In our study, there was no statistically significant difference in 30-day survival rates between patients over 65 and those under 65; however, there was a trend toward higher survival rates in the ECPR group among those under 65. We believe that with a larger sample size, the results could have been more conclusive. ECPR might be more actively considered for younger patients.

This study has several limitations. First, as a single-center, retrospective study, the findings may have limited applicability to other settings or populations. To confirm and broaden the relevance of these results, future research involving larger sample sizes and multi-center studies is necessary. However, due to the nature of the research topic, implementing a prospective design is considered highly challenging. Second, as this was a retrospective observational study comparing two time periods, there may have been residual confounding factors, even after statistical adjustments. In addition to the effect of ECPR, outcomes could have been influenced by cumulative experience over time. To address this, we employed various methods to control for confounders, including IPTW and regression modeling with several risk factors. Third, the follow-up period after cardiac arrest in this study was relatively short. Further research with longer-term follow-ups is necessary to provide more comprehensive insights.

## 5. Conclusions

In patients with refractory OHCA, ECPR may improve neurological outcomes compared to CCPR, although it does not significantly affect 30-day survival. These findings highlight the potential role of ECPR in settings with well-established infrastructure and experienced teams capable of rapid ECMO insertion, such as tertiary care centers and specialized cardiac arrest management units. Implementing ECPR protocols in such healthcare settings could enhance outcomes, particularly for younger patients and those with shorter low flow times. Further studies are necessary to validate these findings and explore the long-term impacts of ECPR while considering its cost-effectiveness and broader applicability.

## Figures and Tables

**Table 1:** Baseline characteristics of all patients.

	Overall Cohort					IPTW				
	Total Patients (n = 58)	ECPR Group (n = 28)	CCPR Group (n = 30)	*p* Value	SMD	Total Patients (n =179)	ECPR Group (n = 78)	CCPR Group (n = 101)	*p* Value	SMD
Age, median (IQR) (yr)	58.0 (50.0–68.0)	55.5 (48.0–65.0)	58.5 (53.0–68.0)	0.121	−0.486	58.0 (50.0–69.0)	54.0 (39.0–65.0)	66.0 (55.0–72.0)	0.001	−0.531
Male, n (%)	46 (79.3)	25 (83.3)	21 (75.0)	0.525	−0.206	142 (79.3)	53 (67.1)	89 (88.1)	0.001	−0.521
Pre-arrest co-morbidities, n (%)										
Coronary artery disease	12 (20.7)	6 (21.4)	6 (20.0)	>0.999	-	30 (16.8)	11 (14.1)	19 (18.8)	0.461	-
Arrythmia	7 (12.1)	3 (10.7)	4 (13.3)	>0.999	-	21 (11.7)	10 (12.8)	11 (10.9)	0.778	-
Heart failure	6 (10.3)	3 (10.7)	3 (10.0)	>0.999	-	15 (8.4)	6 (7.7)	9 (8.9)	0.622	-
Hypertension	23 (39.7)	10 (35.7)	13 (43.3)	0.600	-	66 (36.9)	24 (30.8)	42 (41.6)	0.146	-
Diabetes mellitus	14 (24.1)	9 (32.1)	5 (16.7)	0.224	-	35 (19.6)	18 (23.1)	17 (16.8)	0.304	-
Stroke	3 (5.2)	0 (0)	3 (10.0)	0.238	-	19 (10.6)	0 (0)	19 (18.8)	<0.001	-
Lung disease	1 (1.7)	0 (0)	1 (3.3)	>0.999	-	3 (1.7)	0 (0)	3 (3.0)	0.136	-
Chronic renal disease	4 (6.9)	3 (10.7)	1 (3.3)	0.345	-	8(4.5)	5(6.4)	3 (3.0)	0.423	-
Liver cirrhosis	2 (3.5)	0 (0)	2 (6.7)	0.492	-	5 (2.8)	0 (0)	5 (5.0)	0.060	-
OHCA circumstances, n (%)										
Witness status										
By EMS personnel	10 (17.2)	5 (17.9)	5 (16.7)	>0.999	-	24 (13.4)	10 (12.8)	14 (13.9)	0.852	-
By Layperson	48 (82.8)	23 (82.1)	25 (83.3)	>0.999	-	155 (86.6)	68 (87.2)	87 (86.1)	0.852	-
Bystander CPR	35 (60.3)	18 (64.3)	17 (56.7)	0.600	0.156	98 (54.8)	49 (62.8)	48 (47.5)	0.043	0.311
First documented arrest rhythm (EMS)										
VF/pulseless VT	32 (55.2)	20 (71.4)	12 (40.0)	0.020	0.667	89 (49.7)	65 (83.3)	24 (23.8)	<0.001	1.485
PEA	12 (20.7)	5 (17.9)	7 (23.3)	0.749	-	27 (15.1)	9 (11.5)	18 (17.8)	0.291	-
Asystole	14 (24.1)	3 (10.7)	11 (36.7)	0.031	-	63 (35.2)	4 (5.1)	60 (59.4)	<0.001	-
First documented arrest rhythm (ER)										
VF/pulseless VT	21 (36.2)	18 (64.3)	3 (10.0)	<0.001	-	57 (31.8)	52 (66.7)	9 (8.9)	<0.001	-
PEA	17 (29.3)	8 (28.6)	9 (30.0)	>0.999	-	44 (24.6)	20 (25.6)	24 (23.8)	0.733	-
Asystole	20 (34.5)	2 (7.1)	18 (60.0)	<0.001	-	78 (43.6)	6 (7.7)	72 (71.3)	<0.001	-
Time from collapse to CPR termination, median (IQR) (min)										
No flow time	1.0 (0.0–5.0)	0.0 (0.0–8.0)	3.5 (1.0–5.0)	0.167	0.021	2.0 (0.0–5.0)	0.0 (0.0–6.0)	3.0 (0.0–5.0)	0.967	0.007
Low flow time *	45.5 (32.0–57.0)	45.5 (33.5–63.5)	45.5 (30.0–57.0)	0.581	0.276	44.0 (31.0–57.0)	47.0 (34.0–68.0)	43.0 (30.0–57.0)	0.074	0.287
Pre-hospital CPR time	20.0 (11.0–27.0)	18.5 (11.0–30.0)	20.5 (11.0–26.0)	0.732	-	19.0 (11.0–26.0)	18.0 (12.0–30.0)	20.0 (11.0–24.0)	0.268	-
In-hospital CPR time	28.5 (12.0–34.0)	30.0 (15.0–33.5)	23.5 (12.0–35.0)	0.508	-	27.0 (12.0–34.0)	30.0 (11.0–34.0)	23.0 (12.0–34.0)	0.173	-
Total epinephrine dose, median (IQR) (mg)	9.5 (4.0–12.0)	10.0 (4.5–11.5)	7.5 (4.0–12.0)	0.691	-	7.0 (4.0–11.0)	10.0 (3.0–11.0)	6.0 (5.0–11.0)	0.928	-
Laboratory finding (Initial), median (IQR)										
Lactate (mmol/L) (n = 56)	11.0 (7.0–14.6)	11.0 (6.8–14.9)	11.0 (7.2–14.6)	>0.999	-	10.9 (6.9–15.0)	10.2 (6.9–15.6)	10.9 (7.1–15.0)	0.780	-
pH (n = 56)	7.0 (6.9–7.2)	7.0 (6.9–7.1)	7.1 (6.9–7.2)	0.352	-	7.0 (6.9–7.2)	7.0 (6.9–7.1)	7.1 (7.0–7.2)	0.442	-
Troponin I (ng/mL) (n = 44)	0.1 (0.0–0.5)	0.3 (0.1–0.9)	0.1 (0.0–0.1)	0.023	-	0.1 (0.0–0.4)	0.3 (0.1–0.8)	0.0 (0.0–0.1)	0.082	-
Hospital stay, median (IQR) (day)	4.0 (1.0–16.0)	4.5 (1.5–17.0)	2.5 (1.0–15.0)	0.215	-	4.0 (1.0–16.0)	8.0 (2.0–17.0)	1.0 (1.0–13.0)	0.368	-

Non-normally distributed numerical variables are presented as medians (interquartile ranges) and were tested using the Wilcoxon rank-sum test. Categorical variables are presented as numbers (percentages) and were tested using the chi-square test or Fisher’s exact test. * Low flow time was defined as the combined total duration of pre-hospital and in-hospital CPR. The duration of pre-hospital CPR was recorded in the documentation provided by EMS. For patients undergoing ECPR, the in-hospital CPR duration was defined as the time interval from hospital arrival to the initiation of extracorporeal membrane oxygenation. IPTW, inverse probability of treatment weighting; ECPR, extracorporeal cardiopulmonary resuscitation; CCPR, conventional cardiopulmonary resuscitation; SMD, standardized mean difference; IQR, interquartile range; OHCA, out-of-hospital cardiac arrest; EMS, emergency medical services; CPR, cardiopulmonary resuscitation; VF, ventricular fibrillation; VT, ventricular tachycardia; PEA, pulseless electric activity; ER, emergency room.

## Data Availability

The datasets used and/or analyzed in the current study are available from the corresponding author upon reasonable request. The sources and data used in this study can be deposited publicly.

## Supplementary Materials

The following supporting information can be downloaded at https://www.mdpi.com/article/10.3390/jcm14020513/s1: Table S1: Characteristics of patients according to neurological outcomes; Table S2: Logistic regression analysis of the factors associated with poor neurological outcomes; Figure S1: Kaplan–Meier survival curves for 30-day survival in patients with out-of-hospital cardiac arrest. (a) Patients under 65 years of age; (b) patients 65 years and older.
