# Supplementary material for: Comparative Efficacy of Extracorporeal Versus Conventional Cardiopulmonary Resuscitation in Adult Refractory Out-of-Hospital Cardiac Arrest: A Retrospective Study at a Single Center
Source: J Clin Med. 2025 Jan 15;14(2):513. doi: 10.3390/jcm14020513 (PMC11765839; doi:10.3390/jcm14020513)

Table S1. Characteristics of patients according to neurological outcomes.

	Overall cohort					IPTW				
	Total patients (n = 58)	Good (n = 19)	Poor (n = 39)	*p* value	SMD	Total patients (n = 179)	Good (n = 58)	Poor (n = 121)	*p* value	SMD
CCPR	30 (51.7)	6 (31.6)	24 (61.5)	0.0497	-1.500	101 (56.4)	18 (31.0)	83 (68.6)	< 0.001	-1.666
ECPR	28 (48.3)	13 (68.4)	15 (38.5)		-0.144	78 (43.6)	40 (69.0)	38 (31.4)		0.052
Age, median (IQR) (yr)	58.0 (50.0–68.0)	52.0 (46.0–58.0)	63.0 (54.0–70.0)	0.003	-0.864	58.0 (50.0–69.0)	52.0 (30.0–54.0)	66.0 (57.0–72.0)	< 0.001	-0.690
Male, n (%)	46 (79.3)	14 (73.7)	32 (82.1)	0.502	-0.204	142 (79.3)	41 (70.7)	101 (83.5)	0.043	-0.308
First documented arrest rhythm – VF / pulseless VT, n (%)										
EMS	32 (55.2)	15 (78.9)	17 (43.6)	0.013	0.780	89 (49.7)	45 (77.6)	44 (36.4)	< 0.001	0.285
ER	21 (36.2)	12 (63.2)	9 (23.1)	0.004	0.883	57 (31.8)	35 (60.3)	22 (18.2)	< 0.001	0.505
Time from collapse to CPR termination, median (IQR) (min)										
No flow time	1.0 (0.0–5.0)	0.0 (0.0–4.0)	3.5 (0.0–7.0)	0.023	-0.352	2.0 (0.0–5.0)	0.0 (0.0–1.0)	3.0 (0.0–7.0)	0.120	-0.242

Low flow time*	45.5 (32.0–57.0)	33.0 (20.0–52.0)	52.0 (39.0–61.0)	0.003	-0.815	44.0 (31.0–57.0)	34.0 (25.0–52.0)	47.0 (35.0–61.0)	0.034	-0.365
Pre-hospital CPR time	20.0 (11.0–27.0)	18.0 (13.0–29.0)	21.0 (11.0–27.0)	0.823	-	19.0 (11.0–26.0)	19.0 (13.0–30.0)	20.0 (11.0–26.0)	0.391	-
In-hospital CPR time	28.5 (12.0–34.0)	12.0 (6.0–30.0)	30.0 (20.0–40.0)	0.003	-	27.0 (12.0–34.0)	12.0 (6.0–30.0)	30.0 (20.0–40.0)	0.003	-
Total epinephrine dose, median (IQR) (mg)	9.5 (4.0–12.0)	5.0 (2.0–10.0)	10.0 (5.0–12.0)	0.003	-	7.0 (4.0–11.0)	5.0 (2.0–10.0)	10.0 (5.0–12.0)	0.026	-
Laboratory finding (Initial), median (IQR)										
Lactate (mmol/L) (n = 56)	11.0 (7.0–14.6)	8.9 (5.2–14.2)	12.0 (8.9–15.0)	0.033	-	10.9 (6.9–15.0)	8.9 (5.3–14.2)	11.7 (8.7–15.0)	0.084	-
pH (n = 56)	7.0 (6.9–7.2)	7.1 (6.9–7.3)	7.0 (6.9–7.1)	0.079	-	7.0 (6.9–7.2)	7.1 (6.9–7.3)	7.0 (6.9–7.1)	0.073	-
Troponin I (ng/mL) (n =	0.1 (0.0–0.5)	0.1 (0.0–	0.1 (0.0–	0.913	-	0.1 (0.0–0.4)	0.1 (0.0–	0.1 (0.0–	0.524	-

44)		0.9)	0.4)				0.8)	0.3)		
Hospital stay, median (IQR) (day)	4.0 (1.0–16.0)	16.0 (13.0–29.0)	1.0 (1.0–4.0)	< 0.001	-	4.0 (1.0–16.0)	16.0 (15.0–29.0)	1.0 (1.0–4.0)	< 0.001	-

Non-normally distributed numerical variables are presented as medians (interquartile ranges) and were tested using the Wilcoxon rank-sum test. Categorical variables are presented as numbers (percentages) and were tested using the chi-square test or Fisher's exact test.

*Low flow time was defined as the combined total duration of pre-hospital and in-hospital CPR. The duration of pre-hospital CPR was recorded in the documentation provided by EMS. For patients undergoing ECPR, the in-hospital CPR duration was defined as the time interval from hospital arrival to the initiation of extracorporeal membrane oxygenation.

IPTW, inverse probability of treatment weighting; SMD, standardized mean difference; CCPR, conventional cardiopulmonary resuscitation; ECPR, extracorporeal cardiopulmonary resuscitation; IQR, interquartile range; VF, ventricular fibrillation; VT, ventricular tachycardia; EMS, emergency medical services; ER, emergency room; CPR, cardiopulmonary resuscitation.

Table S2. Logistic regression analysis of the factors associated with poor neurological outcomes.

	Univariable analysis	Multivariable analysis

	OR (95% CI)	*p* value	OR (95% CI)	*p* value
CCPR (Reference: ECPR)	4.81 (2.45–9.45)	< 0.001	4.99 (1.67–14.85)	0.004
Age	1.09 (1.06–1.12)	< 0.001	1.06 (1.02–1.10)	0.002
Male (Reference: Female)	2.13 (1.02–4.48)	0.046	0.08 (0.02–0.38)	0.002
Bystander CPR	0.22 (0.11–0.45)	< 0.001	0.64 (0.16–2.57)	0.534
No flow time	1.14 (1.04–1.26)	0.007	1.10 (0.94–1.29)	0.222
Low flow time*	1.04 (1.02–1.06)	< 0.001	1.06 (1.03–1.09)	< 0.001
VF, pulseless VT as initial rhythm (EMS)	0.18 (0.09–0.36)	< 0.001	0.23 (0.08–0.68)	0.008

*Low flow time was defined as the combined total duration of pre-hospital and in-hospital CPR. The duration of pre-hospital CPR was recorded in the documentation provided by EMS. For patients undergoing ECPR, the in-hospital CPR duration was defined as the time interval from hospital arrival to the initiation of extracorporeal membrane oxygenation.

OR, odds ratio; CI, confidence interval; CCPR, conventional cardiopulmonary resuscitation; ECPR, extracorporeal cardiopulmonary resuscitation; VF, ventricular fibrillation; VT, ventricular tachycardia; EMS, emergency medical services.

Figure S1. Kaplan-Meier survival curves for 30-day survival in patients with out-of-hospital cardiac arrest. (a) Patients under 65 years of age, (b) Patients 65 years and older.

CCPR, conventional cardiopulmonary resuscitation; ECPR, extracorporeal cardiopulmonary resuscitation.

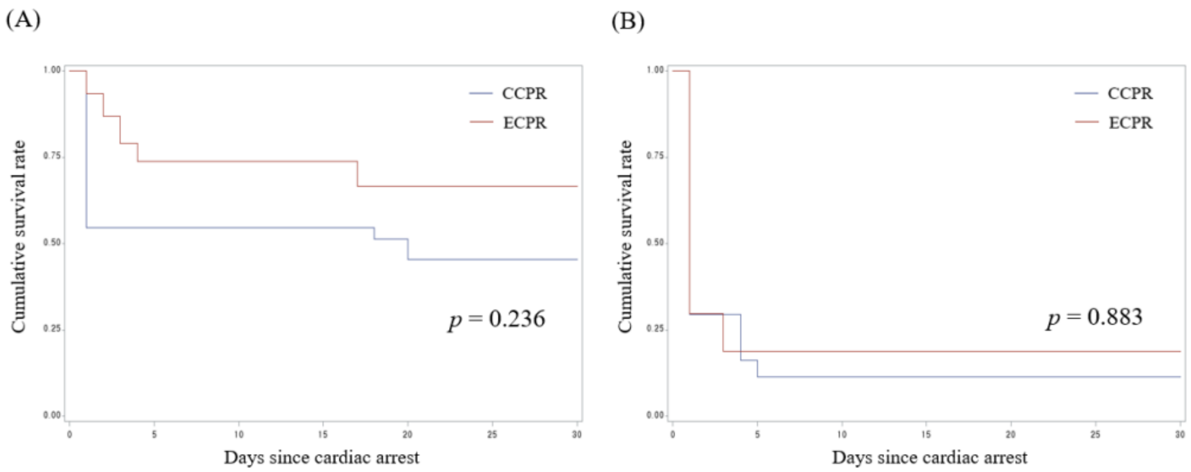

Supplement: Supplementary file 1 [file jcm-14-00513-s001.zip › jcm-3348151-supplementary.pdf]
